# Effect of Wood Species on Lignin-Retaining High-Transmittance Transparent Wood Biocomposites

**DOI:** 10.3390/polym16172493

**Published:** 2024-08-31

**Authors:** Hamza Bradai, Ahmed Koubaa, Jingfa Zhang, Nicole R. Demarquette

**Affiliations:** 1 Forest Research Institute, University of Quebec in Abitibi-Témiscamingue, Rouyn-Noranda, QC J9X 5E4, Canada; hamza.bradai@uqat.ca; 2 State Key Laboratory of Biobased Material and Green Papermaking, Qilu University of Technology Shandong Academy of Sciences, Jinan 250300, China; jingfa.zhang@uqat.ca; 3 Mechanical Engineering Department, École de Technologie Supérieure (ÉTS), Montréal, QC H3C 1K3, Canada; nicoler.demarquette@etsmtl.ca

**Keywords:** biopolymers, lignin modification, transparent wood

## Abstract

This study explores lignin-retaining transparent wood biocomposite production through a lignin-modification process coupled with epoxy resin. The wood’s biopolymer structure, which includes cellulose, hemicellulose, and lignin, is reinforced with the resin through impregnation. This impregnation process involves filling the voids and pores within the wood structure with resin. Once the resin cures, it forms a strong bond with the wood fibers, effectively reinforcing the biopolymer matrix and enhancing the mechanical properties of the resulting biocomposite material. This synergy between the natural biopolymer structure of wood and the synthetic resin impregnation is crucial for achieving the desired optical transparency and mechanical performance in transparent wood. Investigating three distinct wood species allows a comprehensive understanding of the relationship between natural and transparent wood biocomposite properties. The findings unveil promising results, such as remarkable light transmittance (up to 95%) for Aspen transparent wood. Moreover, transparent wood sourced from White Spruce demonstrates excellent stiffness (E = 2450 MPa), surpassing the resin’s Young’s modulus. Also, the resin impregnation enhanced the thermal stability of natural wood. Conversely, transparent wood originating from Larch showcases superior impact resistance. These results reveal a clear correlation between wood characteristics such as density, anatomy, and mechanical properties, and the resulting properties of the transparent wood.

## 1. Introduction

Wood is among the most widely used construction materials due to its excellent properties and renewable aspects [1]. Wood’s treatment has been used for years to incorporate wood in harsher environments. Chemical, heat, and mechanical treatments have been used to enhance wood properties like resistance to fungi, UV light, or moisture [2,3,4,5]. Recently, the focus has been on incorporating an innovative material: transparent wood. This type of material is obtained by chemically treating wood to remove or modify the light-absorbing components, followed by polymer infiltration via vacuum impregnation [6]. The polymers most commonly used in transparent wood are epoxy resin and polymethyl methacrylate [7,8]. These polymers are selected due to their low viscosity, transparency, and compatibility with vacuum impregnation. Moreover, to reduce light scattering, the polymers must have a refractive index close to 1.5 (refractive index of cellulose). The potential use of transparent wood can be seen in different fields, such as transparent building structures, where the natural structure of wood acts as a waveguide, promoting its use for roofing [9,10,11,12,13,14,15,16]. The idea is to replace conventional glass or Plexiglas with a more sustainable material with excellent thermal and mechanical properties on top of its biodegradability [15,17]. Moreover, the functionalization of said transparent wood provides even more interest in its use, such as electromagnetic interference shielding, smart windows, and furniture design with specific luminescent properties [18,19,20,21]. Previous research has demonstrated significant insight in this matter. For instance, Yang et al. [18] demonstrated that introducing Fe_3_O_4_ nanoparticles in transparent wood improves solar-to-thermal conversion and provides a magnetothermal effect on the composite. These results show significant potential for magnetic-to-thermal and solar-to-thermal energy conversion and storage and could efficiently reduce energy consumption. Furthermore, transparent wood has applications beyond the construction field. Removing the light-absorption properties of wood, combined with endless functionalization possibilities, opens the use of transparent wood in electronics, solar cells, and LED production [10,21,22,23,24]. Indeed, Fu et al. [22] produced flexible circuits and sensors by printing bio-based conductive ink on transparent wood films, resulting in sustainable and environmentally friendly wood-based electronics. These films combined properties such as transparency, strength, and flexibility, proving the potential use of transparent wood in electronics. One of the most crucial factors in the final properties of transparent wood is the wood species used in its production. Hardwoods are the most popular wood species used in the recent literature [25,26,27,28]. The reason behind this is the need for differences between earlywood and latewood and their diffuse and porous structure, making the chemical treatment and impregnation process much easier. Low density, which translates into thinner cell walls, is an interesting property that makes light transmission easier and reduces light diffusion in transparent wood [29]. The second important part of transparent wood production is the chemical treatment used. Two main approaches are depicted in the literature.

The first approach is the delignification process, where the idea is to remove lignin completely, which is the source of color and light absorption in the wood [30,31]. The second approach consists of the modification of the lignin [32]. Lignin modification removes the chromophores from its structure without removing its structural function [33,34]. This results in lignin-retaining wood that has better mechanical properties than delignified wood. This difference in mechanical properties could open more fields of transparent wood use in more structural settings. In the past years, different researchers were interested in producing such transparent wood for various purposes and functions, but there was no deep investigation of the effect of wood species and their different properties on the composites. Thus, this paper aims at providing a fundamental understanding of wood species’ effect on transparent wood’s chemical and structural properties. Different wood species and an environmentally friendly chemical treatment are used, and their effect on the different transparent wood properties is studied. This investigation allows a better understanding of the wood, as well as the chemical treatment needed in each application for the transparent wood.

## 2. Materials and Methods

### 2.1. Materials

Three natural wood (NW) species were used in this article: White Spruce [NW-WS], Larch [NW-L], and Aspen [NW-A]. For the lignin-modification process, hydrogen peroxide (H_2_O_2_) (30%), sodium hydroxide (NaOH), and distilled water were used. Trisodium citrate dihydrate was bought from Sigma Aldrich (St.louis, MO, USA). Ethanol, acetone, and sodium hydroxide were purchased from Sigma Aldrich (St.louis, MO, USA). The epoxy resin used was supplied by Composites Envision (Wausau, WI, USA). This resin is a homopolymer (diglycidyl ether of bisphenol) (the epoxy), and the hardener is a mixture of polyetheramine, nonylphenol, and 2-piperazine-1-ylethylamine. 

### 2.2. Lignin Modification

**Figure 1:**
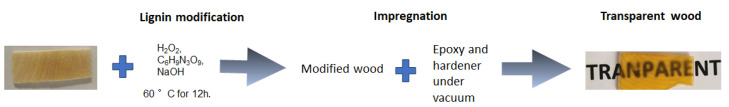
Schematic illustration of the lignin-modification process and transparent wood production process.

Figure 1 presents a schematic illustration of the lignin-modification and transparent wood production process. The natural wood samples with a 2.5 mm average thickness were oven-dried at 103 °C for 24 h before the chemical treatment. The lignin-modification solution used in this process is a mixture of 6 wt% H_2_O_2_, 1 wt% trisodium citrate dihydrate (C_6_H_9_N_3_O_9_), 1 wt% NaOH, and 92 wt% distilled water at 60 °C for 12 h. The bleaching solution is an environmentally friendly alternative to the delignification process. The wood structure and its mechanical properties were preserved by selectively reacting with the lignin chromophores. After chemical treatment, the samples were washed with distilled water and immersed in 50% ethanol and 50% water. The same dehydration process using (ethanol/acetone) was then applied, and the samples were ultimately stored in an acetone solution until they were ready for further use.

### 2.3. Transparent Wood Production 

Figure 1 illustrates that the samples were removed from the acetone solution and subjected to a vacuum cycle to evaporate the acetone. Then, the two-part epoxy resin was mixed with a weight ratio of 100 to 46 (epoxy and hardener). A vacuum cycle was performed to degas the resin before the impregnation process began. The samples were then immersed in an epoxy solution and subjected to at least three cycles of vacuum at 100–200 Pa, each lasting 15 min, to ensure complete impregnation of the wood with epoxy resin. Finally, the impregnated wood (TW) was placed in a rubber container to facilitate its removal after the resin matures. 

### 2.4. Characterization

Fourier transform infrared (FTIR) spectroscopy assessed the surface chemistry of the normal and transparent wood using a Shimadzu IRTracer-100 instrument (Kyoto, Japan). Samples were scanned between 4000 cm^−1^ and 400 cm^−1^ at a resolution of 8 cm^−1^, and an average of 64 scans was recorded. Tensile tests were conducted on a Zwick Universal Testing Machine equipped with a 20 kN load cell (Z020, Zwick Roell Group, Ulm, Germany) to investigate the impact of the transparency treatment on the wood strength of the studied wood species. The tests were conducted according to theAmerican Society for Testing and Materials (ASTM) D3039 [35] on at least five samples of 25 × 50 mm^2^ and a thickness of 2.5 mm and were tested at a crosshead speed of 0.5 mm/s. Impact tests were conducted to evaluate the impact energy of the normal and transparent wood using the Zwick/Roell Izod impact tester with a minimum of five duplicates (BPI-5.5, Zwick Roell Group, Germany). The pendulum utilized has an energy of 2.75 J. The different natural and transparent woods’ surface hardness was assessed through a 560-10D Shore D durometer (Gain Express Holdings, Hong Kong, China) according to the ASTM D2240 [36]. The hardness tests were repeated for at least five samples. The density profiles of the natural wood (NW) and transparent wood (TW) were measured using the tree ring scanner QTRS-01X (QMS, Knoxville, TN, USA). This non-destructive technique uses X-ray beam attenuation through different materials to calculate the density. Natural and transparent wood samples of 25 × 50 mm^2^ and thickness of 2.5 mm were used to determine the density profile. After conditioning, a linear resolution of 20 µm was used to scan the samples in air-dry conditions. Thermogravimetric analyses (TGA) were performed using a TGA Q50 analyzer (TA Instruments, New Castle, DE, USA) under nitrogen on normal and transparent wood samples of the studied wood species to assess their thermal stability. Finally, the transparency tests were performed to assess the transmittance of TW in the visible light range (400–800 nm). The total transmittance was calculated using a UV-visible spectrometer (PuXi TU-1810, Beijing, China) with an integrating sphere to calculate specular and diffuse light according to ASTM D1003 [37]. The sample was placed in front of the sphere entrance, and an incident beam was directed through it. An optical fiber directs the light emitted through the sphere to a spectrometer. The result is a transmittance spectrum in the range of the incident beam (400–800 nm). The anatomy of wood species was studied with confocal microscopy (Keyence, VK-X150 100, Itasca, IL, USA) to assess the effect of natural wood structure on the properties of transparent wood. Furthermore, NW and TW samples were cut in the longitudinal direction. These sections were then coated with a thin layer of gold, essential for observations using scanning electron microscopy (SEM) using a HITACHI tabletop microscope TM4000 (Tokyo, Japan). The coated samples were placed in the microscope, where high-resolution images of the internal structure of the wood were captured. These images allowed the observation of the impregnation effect on the structure of the transparent wood. 

## 3. Results

### 3.1. Physico-Chemical Properties

#### 3.1.1. FTIR Spectroscopy

**Figure 2:**
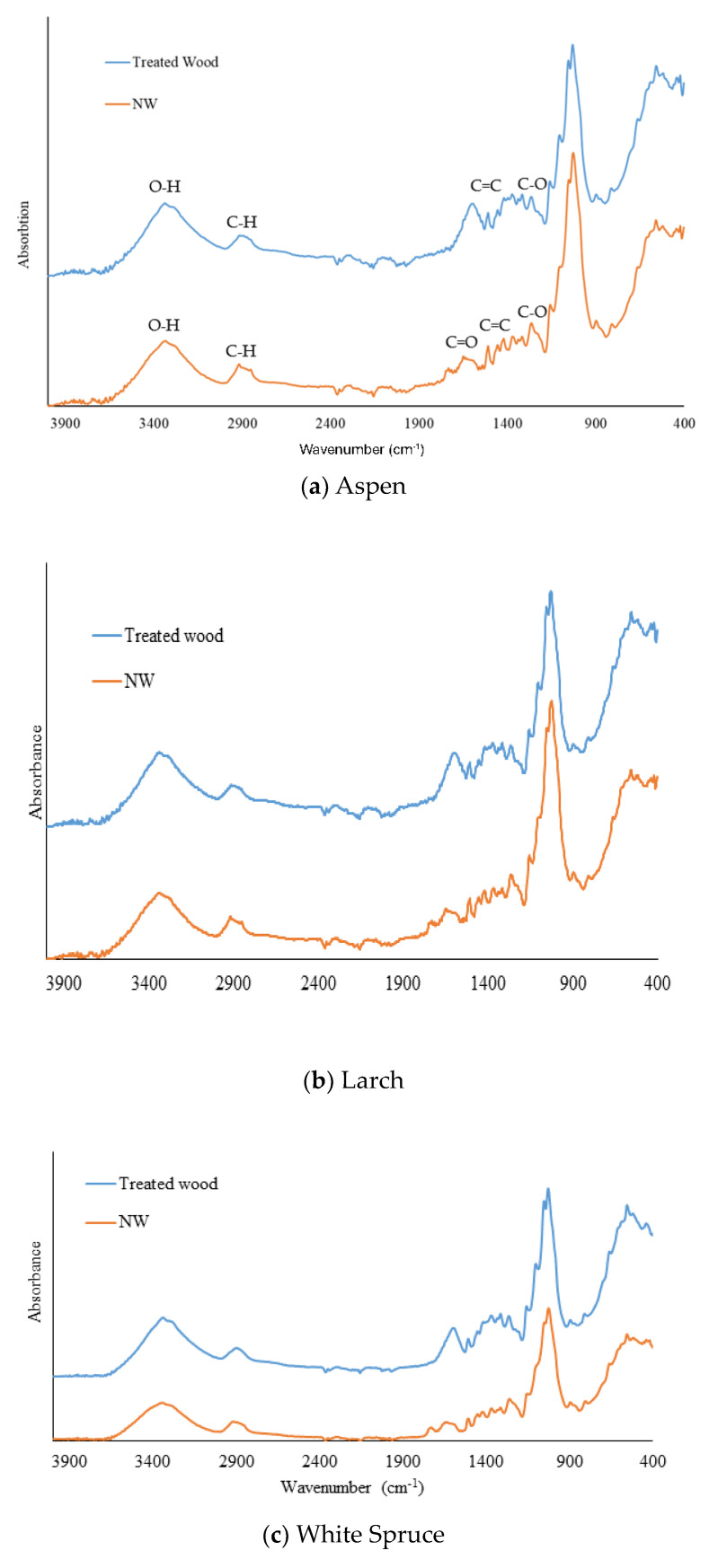
Infrared absorption spectra of (**a**) Aspen, (**b**) Larch, (**c**) White Spruce for natural wood (NW), and lignin-modified wood (treated wood).

Figure 2 shows the FTIR spectra of the natural and lignin-modified wood from the three studied species. The spectra from the three species are similar as the same peaks appear for both treated and normal wood. This similarity is because the same wood components, cellulose, hemicellulose, and lignin, are present in the three species. The band at 3400 cm^−1^ is representative of O-H stretching in hydrogen bonds. The band around 2920 cm^−1^ present in the natural wood represents the stretching of the C-H bond. After the treatment, this band shifts towards 2908 cm^−1^, the same stretching of C-H observed for cellulose. This shift could already start indicating that the lignin is modified, resulting In the shift In the peak for C-H stretching. Peaks in the natural wood and treated wood, such as the one at 1161 cm^−1^, correspond to the guaiacol and syringyl C-H in lignin and glucose ring stretching in cellulose. The peaks at 1269 cm^−1^ and 1319 cm^−1^ are characteristic of the C-O bond for the guaiacyl and syringyl rings, respectively. It is also important to note that the CH_2_ wagging happens at 1319 cm^−1^ [38]. The peak at 1373 cm^−1^ represents the C-H symmetric deformation usually observed in cellulose. The C-H deformation (methyl and methylene) in lignin is present in treated and untreated wood at 1458 cm^−1^, similar to the C-H deformation in cellulose. One of the most characteristic peaks of lignin is present at 1508 cm^−1^; it is associated with the aromatic skeletal vibration and is present in both spectra. On the other hand, the peaks at 1708 cm^−1^ and 1604 representing the C=O (unconjugated) and Aromatic skeletal vibration combined with C=O stretching are only present in the natural wood Spectrum. Thus, the treatment modified the lignin structure without eliminating it. Indeed, the FTIR spectra of the treated wood show a modification of lignin functional groups with the absence of C=O peaks and a shift in C-H stretching peaks, leading to a modification in the chromophore behavior towards light absorption. However, the peaks associated with the aromatic skeleton of lignin confirm that the lignin structure is still intact. Another interesting difference between treated and untreated wood is the peak in 1730 for the carbonyl group, which is representative of holocellulose (hemicellulose and cellulose). This peak is no longer present after lignin modification, meaning that the treatment influenced the hemicellulose content of the treated wood.

#### 3.1.2. Density Profile 

**Figure 3:**
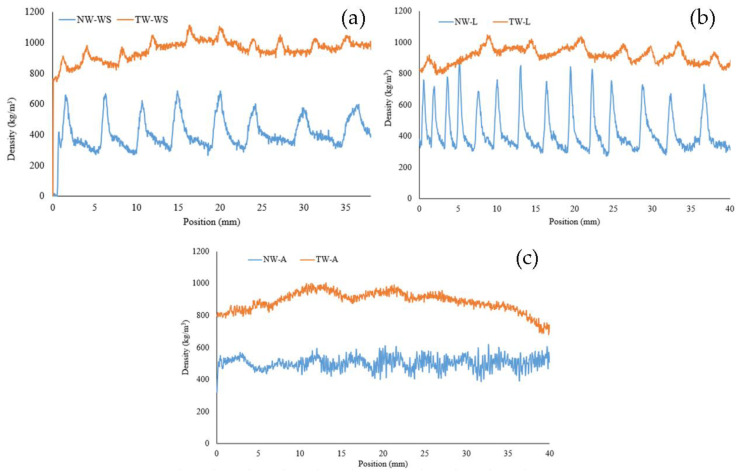
Density profile of different wood species of natural wood (NW) and their transparent wood (TW)equivalent: (**a**) White Spruce (WS), (**b**) Larch (L), and (**c**) Aspen (A).

**Figure 4:**
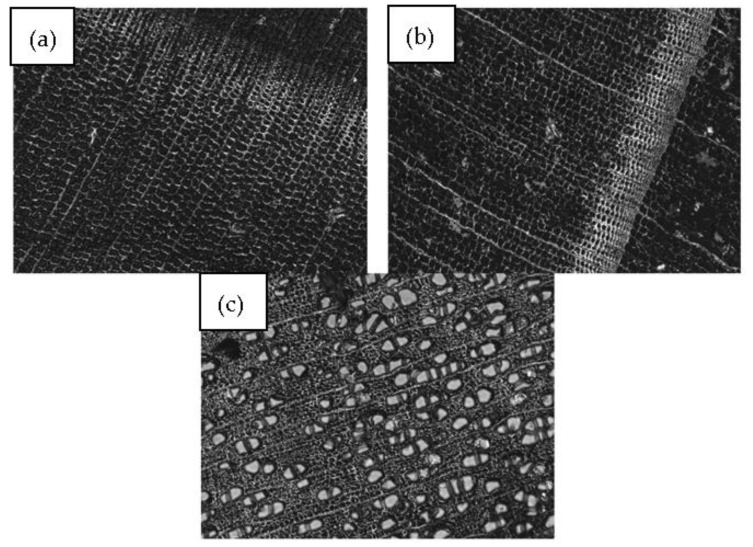
Confocal microscopy images of (**a**) White Spruce, (**b**) Larch, and (**c**) Aspen.

Figure 3 illustrates the density profile of the three different wood species for natural and transparent wood (TW). The main purpose of this test is to assess the impregnation efficacy of epoxy in the wood pores. The density profile varies among the wood species [39]. These differences between the samples are due to wall thickness, porosity, and cell type [40]. The main common factor between the density profiles is the difference between earlywood and latewood, where earlywood has a lower density with thinner cell walls, and latewood is characterized by higher density with thicker cell walls and fewer porosities [41]. It is important to mention that the transition between earlywood and latewood is much more pronounced in White Spruce and Larch than in Aspen (Figure 4). The softwood species (Larch and White Spruce) have a more abrupt earlywood-latewood transition than Aspen, a hardwood with a diffuse pore structure with a gradual earlywood-latewood transition [42,43]. It is also important to note that White Spruce presents a gradual earlywood-latewood transition compared to Larch. 

**Figure 5:**
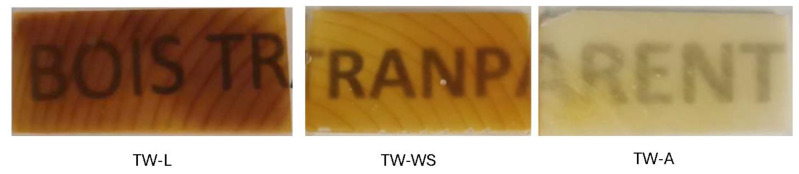
Visual representation of transparent wood from Larch (TW-L), White Spruce (TW-WS), and Aspen (TW-A) showing the wood is darker for higher-density species.

The comparison between the species densities shows that Larch has the largest difference, going from 270 kg/m^3^ to 870 kg/m^3^. On the other hand, NW-A density varied from 322 kg/m^3^ to 637 kg/m^3^. After the impregnation, however, the density profiles have less variability. The high and low values got closer to the mean value, which indicates successful impregnation. The impregnation rate varied within and among species. Although the density after impregnation is more constant, the difference between earlywood and latewood can still be seen with smaller peaks in density. This difference can be seen in the impregnated samples, where the darker areas on the sample represent higher density, as shown in Figure 5. This phenomenon can be explained by the thicker and more condensed latewood slowing down the solution’s diffusion in the cell walls, which leads to a more selective delignification in the earlywood [44,45]. As expected, this was more visible in softwoods, where the difference between earlywood and latewood is more defined than in hardwood. It is to be noted that a longer reaction time of up to 24 h can push the lignin degradation further, resulting in a more uniform density. However, such a reaction would lead to the loss of the structural integrity of the wood, making it impossible to handle the samples.

#### 3.1.3. Thermal Stability

**Figure 6:**
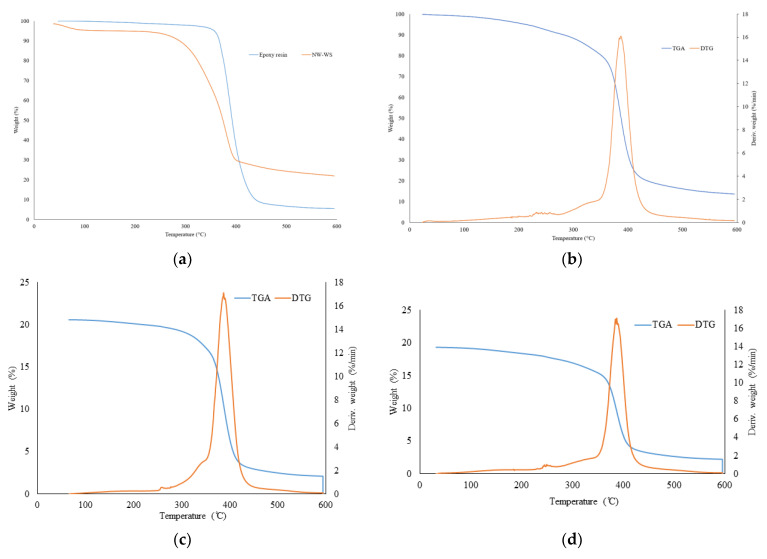
Thermal stability of normal wood (NW) and transparent wood (TW): (**a**) TGA of epoxy resin and NW-WS (White Spruce), (**b**) TGA and DTG of TW-WS, (**c**) TGA and DTG of TW-A (Aspen), (**d**) TGA and DTG of TW-L (Larch).

Figure 6 shows the thermal degradation of the epoxy resin and NW-WS and the different transparent wood from Aspen and Larch. The TGA and DTG spectrums show the same degradation patterns for the different wood species due to the similarities between their cellulose, hemicelluloses, and lignin components. The transparent wood degradation starts at around 100 °C, corresponding to residual water elimination. It is already clear that natural wood has more humidity at this stage. The key takeaway from these curves is the T_max_, which represents the temperature at which maximum degradation occurs, as indicated by the peak in the DTG curve. The maximum temperature (T_max_) for all types of transparent wood is within the range of 400 °C, indicating that the thermal stability of transparent wood is not significantly affected by the wood species. On the other hand, wood thermal degradation peaks at around 350 °C, and the resin starts at 400 °C. Hemicelluloses are the first to degrade at temperatures between 200 °C and 300 °C. Lignin has a wider range of degradation temperatures, from 225 °C to 450 °C. Finally, the degree of polymerization of cellulose begins to decrease at around 200 °C, with full degradation occurring between 300 °C and 450 °C [46,47]. Thus, the thermal stability of the transparent wood is similar to that of the epoxy resin, with a T_max_ of 400 °C [48]. The study of the thermal degradation of transparent wood proves that wood species do not affect the thermal stability of transparent wood. This phenomenon is attributed to the pretreatment of wood before impregnation, which involves the partial removal of lignin and hemicellulose. This process reduces the variations in wood composition between the three species and results in a higher cellulose concentration, the most thermally stable component. Finally, the epoxy resin mentioned in the hardness test provides a protective layer for the wood, leading to higher thermal stability. Table 1 presents the 5% (T_5%_) and 50% (T_50%_) degradation temperatures and the total residue. The results prove an enhanced thermal stability of transparent wood compared to natural wood with an increase in T_5%_ and T_50%_.

#### 3.1.4. Optical Properties

**Figure 7:**
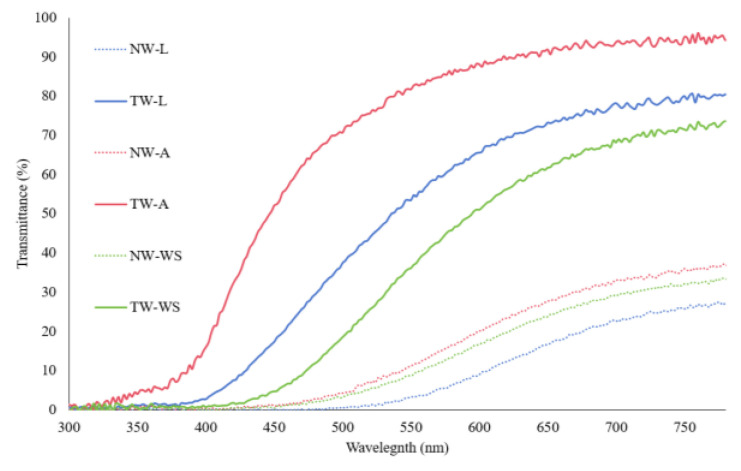
Total transmittance spectra of different wood species: Larch (L), White Spruce (WS) and Aspen (A) for natural wood (NW) and their transparent composites (TW).

Figure 7 illustrates the light-transmittance characteristics of three wood species, both in their natural and transparent forms. It is clear from the transmittance results that the natural wood samples (NW-L, NW-A, and NW-WS) exhibit the lowest transparency. The three samples have the same spectra with minimal values of 10% under 550 nm. These values are slightly higher going to the infrared region (higher wavelengths). The differences in transmittance among the natural wood samples can be correlated to the wood’s physico-chemical properties, such as density, anatomy, and lignin content. Aspen, for example, has a lower density, uniform structure (Figure 4), and lower lignin content than Spruce and Larch [49]. These characteristics enhance light penetration and minimize UV-visible light absorption compared to denser species, such as Larch (NW-L) and White Spruce (NW-WS) [50,51,52]. Indeed, softwoods are known to have a more compact cellular wall and a higher lignin concentration. The same comparison is true for Larch and White Spruce, with Larch being the denser softwood with slightly higher lignin content [53,54,55].

Going from natural wood to transparent wood, the contrast between transmittance is much more substantial. TW-A achieves over 90% transmittance at 750 nm. TW-L and TW-WS also show higher transmittance values than their natural wood counterparts. Indeed, transmittance values for natural and transparent wood go from 25% to 80% for Larch and 32% to 71% for White Spruce. These results confirm a successful chemical treatment and resin impregnation. Moreover, it shows the impact of wood species and their properties on the transparency of the composites. The change for Larch, from least transparent to having a higher transmittance value than TW-WS, can be correlated to the effect of chemical treatment. Indeed, Larch having very low-density earlywood could affect the overall lignin content, thus increasing the transmittance of the transparent wood. 

In conclusion, the difference between the natural wood and transparent wood of different species confirms the substantial effect of wood species and their properties on transparent wood’s optical properties. Moreover, the significant increase in transmittance values validates the success of the lignin-modification treatment and subsequent resin impregnation in enhancing the optical properties of wood [56].

### 3.2. Mechanical Properties 

#### 3.2.1. Tensile Modulus of Elasticity

**Figure 8:**
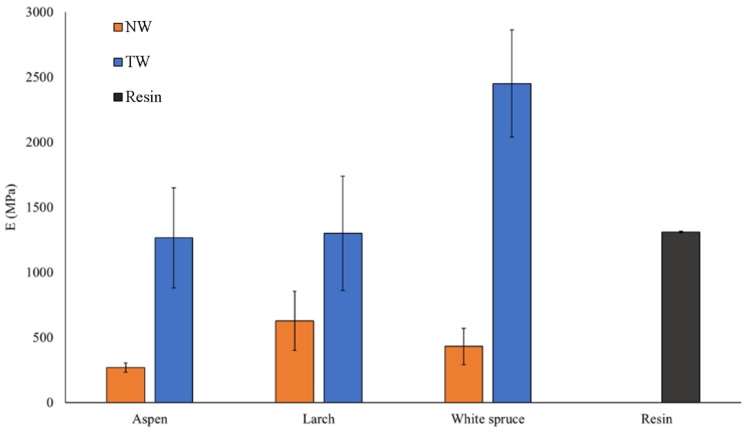
Young’s modulus of natural wood (NW), transparent wood (TW), and pure resin.

**Figure 9:**
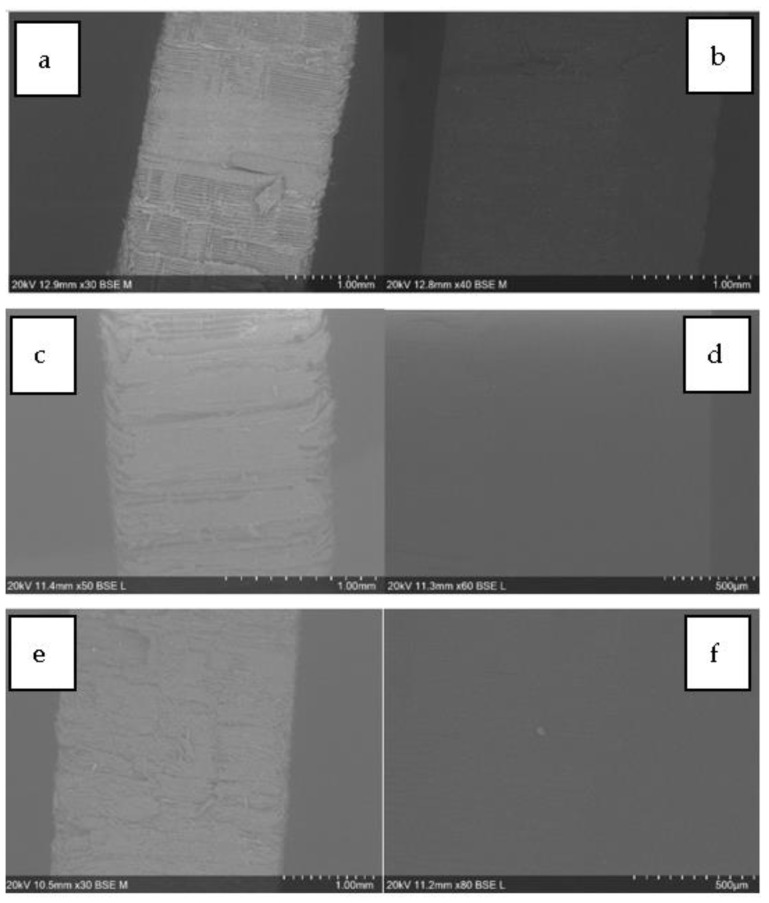
SEM images of natural wood (NW) and transparent wood (TW): (**a**) NW-L (Larch), (**b**) TW-L, (**c**) NW-A (Aspen), (**d**) TW-A, (**e**) NW-WS (White Spruce), and (**f**) TW-WS.

The Young’s modulus (E) of wood, transparent wood, and resin for the three different species is shown in Figure 8. From this graph, wood species impact the modulus [57,58]. Indeed, the lowest E can be attributed to Aspen (NW-A) at 267 MPa, followed by White Spruce (NW-WS) at 430 MPa, and finally, Larch (NW-L) having the highest modulus at 625.5 MPa. These values are mostly related to the mechanical resistance of the wood depending on its density, anatomy, and chemical composition [59,60,61]. Larch, for example, is known to be stiff, strong, and hard compared to Aspen and White Spruce, which are considered soft and have lower strength [62]. However, this trend changes as we move from natural to transparent wood. In fact, with values in the range of 1280 MPa, Larch and Aspen have similar moduli of elasticity. On the other hand, TW-WS has a much higher E of 2450.9 MPa, which is even higher than that of the (1310 MPa). These results can be explained by the different wood species anatomy and density profiles [43,63]. Aspen wood has a low modulus because of its low density and high porosity not typically used for structural products. Therefore, the wood is not a part of the stiffness of the composites; hence, it has a similar modulus then the resin, which governs the composite resistance. Larch, on the other hand, has good mechanical properties as a natural wood [62]. However, the larch density profile has varied from 270 kg/m^3^ to 870 kg/m^3^ (Figure 3). This density difference makes the chemical treatment uneven. When treated, the earlywood, which is less dense, is much more delignified than the latewood, which is denser. The wood structure is weakened, resulting in poor mechanical properties, such as the modulus (E) in the composites. Finally, the White Spruce exhibits decent mechanical properties and has lower-density variation, making it more suitable for a balanced chemical treatment overall. This results in effective resin impregnation within the well-maintained wood structure. As a result, TW-WS has a modulus of 2451 MPa, which is higher than the resin itself. The white Spruce exhibited the highest increase in modulus from natural wood to transparent wood, with values of 430 MPa and 2451 MPa, respectively. Tensile test results indicate that the composites’ mechanical properties vary among the wood species. Although the mechanical properties of natural wood are important, wood anatomy, density, and chemical treatment affect the stiffness of transparent wood. The SEM images (Figure 9) show the effect of the impregnation on the wood structure. These images confirm the successful impregnation process since the voids previously visible in the NW (Figure 9a,c,e) are no longer present in the TW (Figure 9b,d,f). These observations further explain the enhancement in the mechanical properties of transparent wood compared to natural wood. Indeed, after impregnation, it is harder to differentiate the wood from the resin because of the adhesion between the two components of the composite. 

**Figure 10:**
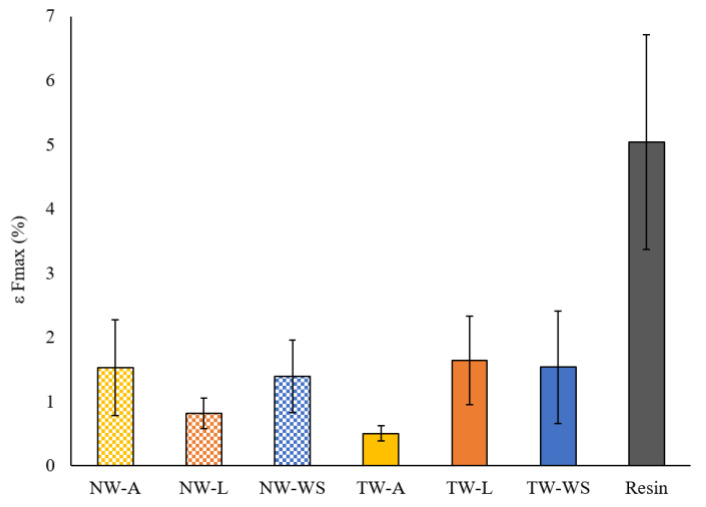
Elongation at F_max_ (ε_Fmax_) of natural wood, transparent wood, and resin.

The elongation at F_max_ (ε_Fmax_) results are summarized in Figure 10 for the different natural and transparent woods. Comparing natural woods, it is evident that wood species influences the ε_Fmax_, with Aspen presenting the highest elongation, followed by White Spruce and Larch. However, the TWs show different results. Indeed, TW-L and TW-WS show higher elongation than TW-A. Finally, the resin shows a much higher ε_Fmax_ compared to the composites. These results reinforce the assumption that there is a weakness in the resin/wood interface. Moreover, TW has similar or lower ε_Fmax_ than NW due to the weakening of the wood after the chemical treatment. Since lignin acts as a glue for wood fibers, removing it reduces the wood’s ability to stretch before breaking. It can be concluded that the wood species influences ε_Fmax_. However, more tests are needed to confirm if these effects are due to the wood’s structure or the wood/resin interface. 

#### 3.2.2. Impact Resistance

**Figure 11:**
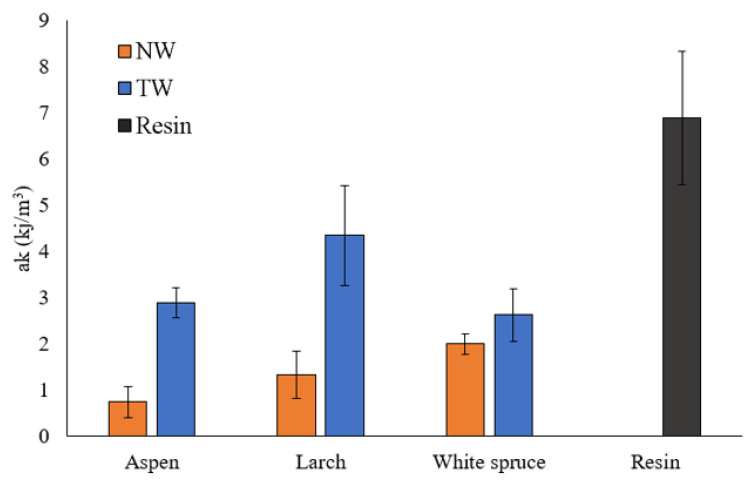
Impact resistance of natural wood (NW), transparent wood (TW), and resin.

Figure 11 summarizes the impact strength (ak) for transparent wood, natural wood, and epoxy resin. Comparing natural and transparent wood, it is evident that adding resin improves the impact resistance of the samples. Indeed, the ak goes from 2 kJ/m^2^ for the White Spruce to 2.63 kJ/m^2^. The same result is true for Larch and Aspen, with values going from 1.33 kJ/m^2^ and 0.74 kJ/m^2^ for natural wood to 4.34 kJ/m^2^ and 2.89 kJ/m^2^ for transparent wood, respectively. On the other hand, the transparent wood has a lower impact resistance than the resin itself. This result is expected because, despite good adhesion and interaction between the epoxy resin and wood sample, there may still be gaps between the cell wall and the resin, leading to crack propagation. This ultimately results in a lower ak for transparent wood (4.34 kJ/m^2^ at maximum) compared to epoxy resin, which has an impact strength of 6.89 kJ/m^2^. These results indicate that the wood species affects the sample’s impact strength. Indeed, TW-L had a higher ak (4.34 kJ/m^3^) than Aspen and White Spruce (2.89 and 2.63 kJ/m^3^, respectively). These results prove that the wood species used impacts the transparent wood produced. Larch wood, known to have high mechanical properties and shock resistance, is used in construction for rough dimensions, resulting in a transparent wood with higher impact resistance. Another explanation for the Larch transparent wood having such a high impact resistance could be correlated to its density profile. Indeed, Larch has highly dense latewood that could retain its structural strength even after lignin modification [64]. These high-density areas could provide more resistance to fracture dispersion, leading to a higher overall impact resistance for the TW-L [65].

#### 3.2.3. Hardness

**Figure 12:**
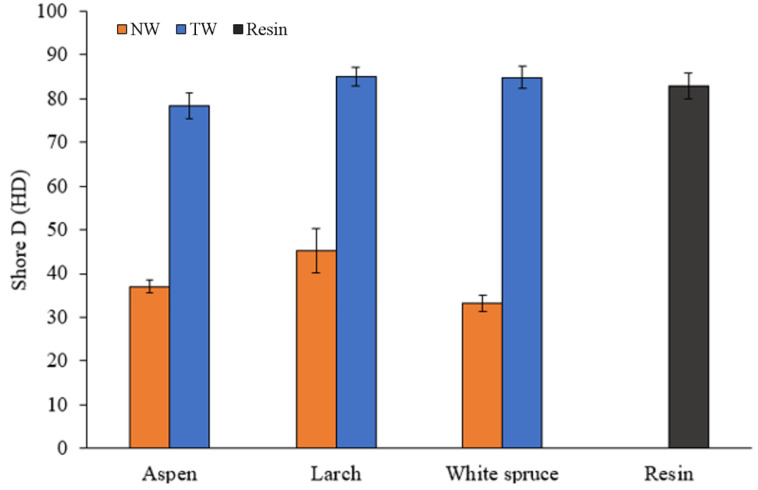
Surface hardness of different wood species: natural woods (NW), transparent woods (TW) and resin.

The Shore D hardness test measures the resistance to penetration of hard rubbers and plastics. Figure 12 displays the surface hardness results for various natural, transparent, and resin woods. The species of wood used has an impact on the Shore D of natural wood. Larch has the highest properties with hardness, followed by Aspen, then White Spruce with hardness values of 45.2 HD, 37 HD, and 33.2 HD, respectively. It is important to note that Larch exhibited the highest variability in hardness, primarily due to significant differences between earlywood and latewood densities [57,66]. The species has less impact on the transparent wood hardness. TW-A has the lowest Shore D value at 78.42, while TW-L and TW-WS had similar Shore D values, with 85.02 and 84.86, respectively. All the samples had similar hardness values to the resin, which has an 82.92 HD. A protective resin layer on the composite surface is among the plausible explanations. However, these results are positive, indicating that the impregnation process doesn’t have a negative impact on the resin properties. Additionally, the sturdy resin layer on the sample’s surface indicates a positive interaction between the wood and the epoxy resin.

## 4. Conclusions

In conclusion, lignin-modified transparent wood was successfully fabricated with three different wood species, two softwoods, and one hardwood. This work aimed to help understand the effect that the wood species and its properties have on transparent wood. The wood species influenced Young’s modulus and impact strength from the mechanical results. Wood’s mechanical properties, anatomy, and density profiles impacted the transparent composite properties. Wood’s profile density and anatomy impacted the fabrication process, such as the chemical treatment and the impregnation process. Wood with lower density and less variation in its density profile facilitates the fabrication process with easier treatment and impregnation. However, species like Aspen with low density and low mechanical properties resulted in transparent wood with lower mechanical properties. On the other hand, White Spruce resulted in a Young’s modulus that was higher than the resin itself. It is also important to note that the high variability within the density profiles weakens the wood during the chemical treatment, thus resulting in lower transparent wood’s mechanical properties. Regarding optical properties, the transmittance spectra show a clear effect of wood species on the transparency of the resulting composites. A low density enhances transparency, with TW-A showing the least absorbance throughout the visible light spectrum. Notably, a maximum value of 95% transmittance for an average of 2.5 mm thickness and lignin modification is very high compared to more invasive treatments like delignification. Wood species do not influence surface hardness and thermal stability. The improvement of the mechanical and thermal properties is attributed to the resin impregnation. The lignin-modification process is mainly used to reduce light absorption in UV-visible light. However, the treatment affects the chemical structure of lignin and removes parts of the hemicellulose, weakening the mechanical properties. Therefore, the epoxy resin is impregnated in the wood pores and fiber lumen to enhance these properties, resulting in stiffer transparent wood. 

## Figures and Tables

**Table 1:** Degradation temperature at 5% and 50% weight lost and residue for NW-WS, resin, and TW-WS, TW-A, and TW-L.

Sample	T_5%_(°C)	T_50%_(°C)	Residue (%)
NW-WS	80.3	374.2	22.0
NW-A	72.7	322.5	16.8
NW-L	75.3	348.5	15.6
Resin	357.0	392.5	5.5
TW-WS	212.3	387.6	13.7
TW-A	272.0	387.9	10.2
TW-L	197.4	386.8	11.5

## Data Availability

Data will be available upon request.
